# Transfusion transmitted infections: How many more?

**DOI:** 10.4103/0973-6247.67017

**Published:** 2010-07

**Authors:** Nabajyoti Choudhury

**Affiliations:** *Editor, Asian Journal of Transfusion Science, India*

Transfusion transmitted infections (TTI) are a great concern of safety for patients. Since the starting of blood transfusion scientifically in the early 1940s, various transfusion associated problems have come to the forefront for the scientific community. These include TTI, alloimmunization to various blood components, issues related to cold chain maintenance, platelet refractoriness, transfusion (iron) overload, transfusion associated graft versus host diseases (GvHD), immunomodulatory effects, etc. However, TTI was first observed in the process of blood transfusion in the late 1940s. Till early 1970s, blood bank personnel were only concentrating on a few blood borne infections like syphilis and serum hepatitis by “Australia antigens”. However, the scientific community was well aware that there would be multiple agents.

In the last 40 years, the scientific community has noted that numerous viral, bacterial and parasitic infectious agents are involved as hurdles in blood safety to patients. There are even some infections which have been proved to be transfusion transmitted and we, transfusion workers, were not aware of these infections. This editorial is in relation to two manuscripts published in the same issue of this journal. One is a review article on transfusion associated parasitic infections and the other one is on parvovirus B 19.

The magnitude of the TTI varies from country to country depending on TTIs’ loads in that particular population from where blood units are sourced. Multiple measures are taken to minimize TTI transmission in the respective population. These strategies may be targeted to prevent transfusion-transmitted diseases in that country. There is a risk of 1–2 per 1000 recipients receiving contaminated blood with viral, bacterial or parasitic agents. However, there is 50% risk of serious morbidity and mortality for the patients if blood transfusion is not done or undertaken. Viral infections assume a great importance in transfusion associated mortality and morbidity in patients. Majority of the problems are due to prevalence of asymptomatic carriers in the society, as well as, blood donations during the window period of infections. Concealing of medical history by captive, paid or professional blood donors, who widely exist in developing countries, also pose a great threat to safe blood supply. There is a long list of viruses, parasites and bacteria, which can be transmitted through blood transfusions. Among them, important transfusion-transmitted viruses are human immunodeficiency virus (HIV-I/II), hepatitis B virus (HBV), hepatitis C virus (HCV), syphilis infection by spirochytes and transfusion associated malaria infection.

We are well aware of the common TTI mentioned above in South Asian countries. However, there are infections which are not routinely screened in blood transfusion services (BTS) in this part of the world. They are a threat to blood safety. There are many infections which can be transmitted through blood transfusions but it is not possible to screen for these rare or very common diseases. Let us take the example of cytomegalovirus (CMV) infection. The prevalence of anti CMV (IgG) in Indian subcontinent is about 95%. About 5% of screened donor population have IgM antibody which carries eminent threat of transmitting CMV infection to immune-compromised and newborn patients. Due to the wide prevalence of CMV infection in this population, it is not possible to screen blood donors for this infection. The practical solution is to supply leukoreduced (by 3^rd^/4^th^ generation filters) blood products to the needy patients.

Similar observation has been made in one of the published articles in this issue on Parvo virus B-19. There is a paucity of reports on B-19 infection in Indian subcontinent. This study was carried on 1000 healthy voluntary blood donors from North India and 399 (39.9%) showed evidence of B-19 infection (IgG). There was evidence that as age advances, the prevalence of infection rises. The prevalence of this infection is high in blood donors with poor standard of living, poor socioeconomic status, low educational level, overcrowding and with poor housing conditions (under publication). Now, as a BTS worker, what can we do for B-19 infection? Not much because 40% prevalence in donor population is too high to frame any selection criteria. Moreover, usually this infection is not fatal in immune-competent patients, and once the antibody is developed it is a protective antibody. It is definitely a transfusion hazard but with this background we may not suggest regular screening of blood donors for B-19 in this editorial.

Transfusion associated leishmaniasis is an important TTI in South Asia. Authors of the review article in this issue of AJTS have claimed that there are 10 cases of transfusion associated leishmaniasis reported from Asia and Europe and one case report was from India. Average incubation period of leishmaniasis was reported to be 7.4 (plus and minus) 5 months. There are no recommendations for blood donor screening criteria and there are no screening tests available if someone thinks about that. However, author of this article claims that rapid screening tests are available for blood donor screening at a “very economical price”. It will be a matter of debate if we really want to introduce leishmaniasis screening in the subcontinent. We need more scientific evidence to establish the risk as per prevalence in the donor population and then suggest a screening test as per sensitivity and specificity of the test.

One emerging infection, i.e. chinkungunya virus (CHIKV), is threatening BTS in South Asian countries. Recently, large CHIKV outbreaks originating in Kenya have spread to islands of the Indian Ocean and parts of India, Southeast Asia, and Europe. Transfusion associated CHIKV is a problem of high population infection incidence during outbreaks and the high-titer viremia lasts for approximately 6 days. Estimated transfusion risk is about 150 per 10,000 transfusions during the outbreak. Transfusion associated CHIKV is not yet established except a case in France where a nurse got infected after coming in contact with infected blood from patient. However, there are multiple assumptions that CHIKV could be transmitted through transfusion. Possible measures to prevent transfusion associated CHIKV are to defer symptomatic blood donors, discontinue blood collection in the affected area and CHIKV nucleic acid screening of donation. Screening for CHIKV is still not possible. Due to sporadic and low prevalence infections, it is more practical to defer symptomatic blood donors for about 12 weeks though this time period is not based on scientific study.

Danger of transfusion associated West Nile virus (WNV) was observed in USA in 2002 and screening test was implemented in 2003. The prevalence of screening test positivity is about 0.001% (818 positive in 6 million units) in the USA. About 80% of viremic donors are asymptomatic and viremia lasts for 6.5 days. There is no study to prove the transfusion associated WNV in Indian subcontinent. Contrary to common belief, WNV, a mild, non-fatal dengue like illness in humans, is highly prevalent in India. Febrile illness in epidemic form and clinically overt encephalitis cases were observed in Udaipur area of Rajasthan, Buldhana, Marathwada, and Khandesh districts of Maharashtra province of India. WNV neutralizing antibodies (about 20–30%) have been detected in human sera collected from Tamil Nadu, Karnataka, Andhra Pradesh, Maharashtra, Gujarat, Madhya Pradesh, Orissa, and Rajasthan. We will not be able to comment on screening for WNV in South Asia in the absence of strong scientific study on prevalence, transfusion associated transmission and disease outcome.

Severe acute respiratory syndrome (SARS) is caused by the coronavirus which caused a major outbreak in 2003 in Asia and secondary cases elsewhere in the world. There is no direct evidence of transfusion associated SARS; however, there is a theoretical risk through transfusion of labile blood component. Low viremia remains for 10 days after the onset of symptoms. The Department of Blood Safety and Clinical Technology of World Health Organization recommended deferring suspected blood donors and asked blood donors to report back if suffering from SARS within 1 month. It also recommended to follow up the recipients of blood and components from donors who are present with symptoms, within 1 month of transfusion. However, it seems that there is no requirement of sounding any alert for donor screening for SARS.

The list of new transfusion transmitted disease threat does not end here. Many blood borne viral, parasitic, and bacterial agents may be transmitted through transfusion. Many more may emerge in future. However, it will not be possible to screen for all the diseases. We may have to be more selective about diseases which are persistent in human beings for a longer time like hapatotropic viruses, pathological agents lodged at RE system, in blood cells, etc. How do we get rid of these new threats to blood supply?; Probably, we have to stick to age old golden rule of sourcing blood from voluntary repeat donors and regular screening of blood units with most sensitive available/affordable techniques. One of the most important weapons we do not use against TTI in South Asia is the hemovigilance. Once we transfuse patients with blood and components, there is no system to follow up for any long-term post-transfusion effects. Barring a few cases of litigations in consumer court, our patient population usually does not report back. There are multiple reasons for which the patients do not report back. Many a times, the patient expires due to the underlying pathology or it is not diagnosed as a post-transfusion complication or there is no awareness among the general practitioners about these problems or patients are simply lost to follow up. If it is diagnosed also, it is often difficult to prove as a post-transfusion complication. It is the need of the hour that the National Blood Transfusion Council (NBTC) should take initiative to start “meaningful” hemovigilance system for Indian BTS, which has not taken off for the last couple of years. NBTC could probably implement hemovigilance through Technical Resource Group (Blood Safety) which is supposed to be more technically involved.

Other modes for pathogen reduction/elimination are leuko reduction, using diversion pouch in blood collection, aseptic blood collection, etc. There is a new technology to destroy/inactivate pathological substances in labile blood components using chemicals/UV lights inside bags. Many more new technologies are expected to come up. However, we are far from achieving zero risk transfusion from TTI transmission is concerned. Till then, collection of blood units form low risk voluntary repeat blood donors and testing with most sensitive tests available are important. Lastly, we must concentrate on improving the overall quality standard of our blood banks. It can be archived by implementing Total Quality Management in most of our blood banks and also by obtaining accreditation by competent authority in this process.

